# Long noncoding RNA H19 indicates a poor prognosis of colorectal cancer and promotes tumor growth by recruiting and binding to eIF4A3

**DOI:** 10.18632/oncotarget.8063

**Published:** 2016-03-14

**Authors:** Dong Han, Xu Gao, Meng Wang, Yu Qiao, Ya Xu, Jing Yang, Nazhen Dong, Jun He, Qian Sun, Guixiang Lv, Changqing Xu, Ji Tao, Ning Ma

**Affiliations:** ^1^ Department of Biochemistry and Molecular Biology, Harbin Medical University, Harbin, China; ^2^ Heilongjiang Academy of Medical Sciences, Harbin, China; ^3^ Department of General Surgery, the Second Affiliated Hospital of Harbin Medical University, Harbin, China; ^4^ Department of Pathophysiology, Harbin Medical University, Harbin, China; ^5^ Department of Gastrointestinal Medical Oncology, the Affiliated Tumor Hospital of Harbin Medical University, Harbin, China

**Keywords:** colorectal cancer, long noncoding RNA, H19, poor prognosis, cell proliferation

## Abstract

The overall biological role and clinical significance of long non-coding RNA H19 in colorectal cancer (CRC) remain largely unknown. Here, we firstly report that the lncRNA H19 recruits eIF4A3 and promotes the CRC cell proliferation. We observed higher expression of H19 was significantly correlated with tumor differentiation and advanced TNM stage in a cohort of 83 CRC patients. Multivariate analyses revealed that expression of H19 served as an independent predictor for overall survival and disease-free survival. Further experiments revealed that overexpression of H19 promoted the proliferation of CRC cells, while depletion of H19 inhibited cell viability and induced growth arrest. Moreover, expression profile data showed that H19 upregulated a series of cell-cycle genes. Using bioinformatics prediction and RNA immunoprecipitation assays, we identified eIF4A3 as an RNA-binding protein that binds to H19. We confirmed that combining eIF4A3 with H19 obstructed the recruitment of eIF4A3 to the cell-cycle gene mRNA. Our results suggest that H19, as a growth regulator, could serve as a candidate prognostic biomarker and target for new therapies in human CRC.

## INTRODUCTION

Colorectal cancer (CRC) is one of the most common malignancies in the world. More than 1 million individuals will develop CRC each year, and the disease-specific mortality rate is nearly 33% in the developed world [1]. The initiation of CRC is a complex biological process that involves multiple genomic and epigenomic alterations [2]. Although encouraging progress in diagnosis and cancer therapy has been achieved in the past decade, the overall survival rate remains unfavorable [3]. A growing incidence and poor outcome of CRC has intensified attempts toward unraveling the underlying pathological mechanisms of CRC progression. Therefore, the development of novel and effective strategies is an urgent need for the early diagnosis and treatment of CRC.

It is well known that protein-coding genes account for only 2% of the total genome, whereas the vast majority of the human genome can be transcribed into noncoding RNAs [4–6]. Among them are long noncoding RNAs (lncRNAs), which are more than 200 nt in length and have limited or no protein-coding capacity. LncRNAs are often expressed in a disease-, tissue-, or developmental stage-specific manner [7, 8]. Multiple lines of evidence have revealed that lncRNAs have oncogenic and tumor suppressor roles in tumorigenesis [9]. The long non-coding RNA (lncRNA) H19 gene is located on chromosome 11 in humans and is a maternally expressed imprinted gene that plays a vital role in mammalian development [10, 11]. Although H19 has been intensively studied in genomic imprinting, the pathological function of H19 as a non-coding RNA has only recently begun to be elucidated [12]. Emerging evidence shows that expression of H19 is upregulated in many cancers, including CRC [13, 14], hepatocellular carcinoma [15, 16], esophageal cancer [17], ovarian cancer [18], breast cancer [19–21] and bladder cancer [22, 23], with or without the loss of imprinting. The overexpression of H19 in cancer tissue hints for its oncogenic function, but the exact underlying mechanism is still not clear.

Previously, the oncogenic role of H19 in CRC was reported to be associated with its function as the precursor of miR-675 [14]. Although some of the H19 target genes have been identified, the mechanism by which H19 binds to these target genes is also still unclear [24]. It is believed that long noncoding RNA can bind directly with its target genes or indirectly through some of the RNA interaction proteins [6, 25, 26]. In our current study, we seek to determine the potential binding proteins of H19 and the mechanisms of dysregulated H19 in colorectal carcinogenesis. We found that upregulation of H19 was a characteristic molecular change and could serve as an independent predictor for overall survival in CRC. Moreover, H19 could increase cell growth by accelerating cell cycle progression. In addition, we demonstrated that H19 could bind to eIF4A3, a core exon junction complex (EJC) component that is loaded onto mRNAs by pre-mRNA splicing, thus regulating the expression level of cell cycle-associated factors and contributing to promoting CRC cell proliferation. Our findings suggest that lncRNA H19 might represent a novel indicator of poor prognosis in CRC and could be a potential therapeutic target for diagnosis and gene therapy.

## RESULTS

### Expression of H19 is upregulated in CRC tissues

**Figure 1:**
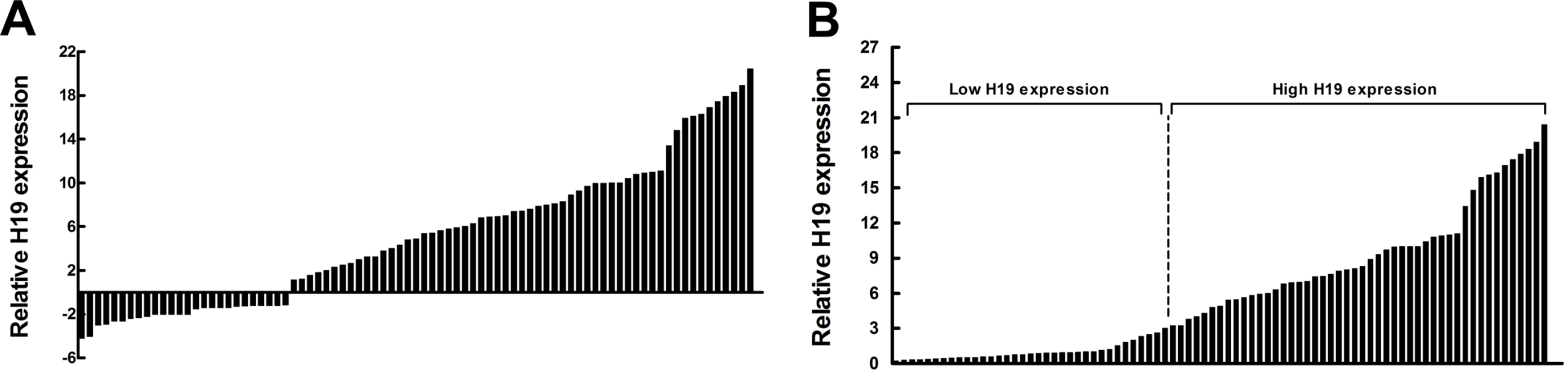
Relative H19 expression in human CRC tissues (**A**) Relative expression of H19 in CRC tissue (*N* = 83) compared with corresponding non-tumor tissue (*N* = 83). H19 expression was examined by qPCR and normalized to β-actin expression. The results are presented as the fold-change in tumor tissue relative to normal tissue. (**B**) H19 expression was classified into two groups.

The level of H19 was detected in 83 paired CRC tissues and adjacent normal tissues by qRT-PCR and normalized to β-actin. Furthermore, expression of H19 was significantly upregulated in 68.7% (57 of 83) cancerous tissues compared with normal counterparts (*P* < 0.01) (Figure 1A). To assess the correlation of H19 expression with clinicopathologic data, according to the relative H19 expression in tumor tissues, the 83 CRC patients were classified into two groups: the relative high group (*n* = 48, fold change ≥ 3) and the relative low group (*n* = 35, fold change ≤ 3) (Figure 1B).

### Overexpression of H19 is associated with tumor differentiation, TNM stage and poor prognosis of CRC

To further understand the significance of H19 overexpression in colorectal cancer, we set out to identify the potential associations between H19 expression and patients' clinicopathological features. Several clinicopathological features of 83 CRC patients were summarized in Table 1. The detailed relationships between the H19 expression status and clinicopathological variables of 83 patients are also shown in Table 1. Noticeably, high expression of H19 in CRC had a significant correlation with the tumor differentiation (*P* = 0.006) and advanced TNM stage (*P* = 0.026). However, H19 expression was not associated with other parameters, such as age (*P* = 0.415) and gender (*P* = 0.163), in CRC (Table 1).

**Table 1:** Correlation between the H19 expression and clinicopathological characteristics of CRC

Characteristic	Low h19 expression(*n* = 35)	High H19 expression(*n* = 48)	*P*
Age (years)			0.415
< 60	18	29	
≥ 60	17	19	
Sex			0.163
Male	20	20	
Female	15	28	
Tumour site			0.384
Rectum	12	21	
Colon	23	27	
Tumour histology			0.245
Adenocarcinoma	22	24	
Mucinous adenocarcinoma	13	24	
Tumour differentiation			0.006*
Well/moderate	19	12	
Poor	16	36	
T stage			0.713
T2/3	16	20	
T4	19	28	
N stage			0.52
N0	15	24	
N1/2	20	24	
TNM stage			0.026*
I + II	21	17	
III + IV	14	31	
Lymph vascular invasion			0.129
Absence	19	18	
Presence	16	30	
Distant metastasis			0.16
Absence	15	28	
Presence	20	20	

**Figure 2:**
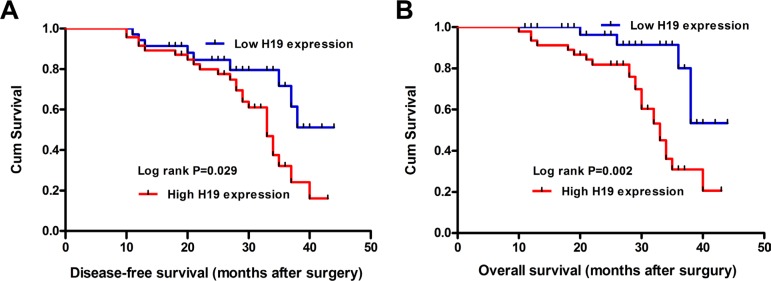
The correlation between H19 expression and the DFS or OS of CRC patients Kaplan-Meier analysis of disease-free survival (**A**) or overall survival (**B**) was analyzed according to the H19 expression levels.

To determine the relationship between H19 expression and CRC patients' prognosis, we attempted to evaluate the correlation between H19 expression and clinical outcomes. Kaplan-Meier analysis and the log-rank test were used to evaluate the effects of H19 expression and the clinicopathological characteristics on disease-free survival (DFS) and overall survival (OS). The results showed that 4-years disease-free survival (DFS) is 17.8% for high H19 expression and 45.1% for low H19 expression. The median survival time is 28 months for high H19 expression and 43 months for low H19 expression (Figure 2A, Log rank *P* = 0.029). Moreover, the 4-years overall survival is 19.3% for high H19 expression and 47.1% for low H19 expression. The median survival time is 34 months for high H19 expression and 45 months for low H19 expression (Figure 2B, Log rank *P* = 0.002).

To further assess whether H19 expression can be identified as a prognostic predictor for CRC patients, univariate and multivariate survival analyses (Cox proportional hazards regression model) were performed. Univariate analyses of clinical variables that were considered to be potential predictors of survival are shown in Table 2. Further analysis in a multivariate Cox proportional hazards model showed that H19 expression, together with TNM stage and tumor differentiation, were strongly associated with DFS (*P* = 0.018, *P* = 0.007, *P* = 0.009, respectively). At the same time, H19 expression, TNM stage and tumor differentiation was also significantly correlated with OS in our study cohort (*P* = 0.006, *P* = 0.008, *P* = 0.006, respectively). The results revealed that H19 expression was an independent prognostic indicator for DFS (HR = 1.521, 95% CI, 1.303–1.896; *P* = 0.018) and OS (HR = 1.433, 95% CI, 1.239–1.786; *P* = 0.006) in patients with CRC (Table 2).

**Table 2:** Univariate and multivariate Cox regression analysis H19 for DFS or OS of patients in study cohort (*n* = 83)

Variables	DFS			OS		
	HR	95% CI	*P* value	HR	95% CI	*P* value
**Univariate analysis**						
Age (< 60 years vs. ≥ 60 years)	1.2	0.74–1.95	0.455	1.3	0.98–2.62	0.061
Gender (Male vs. Female)	1.419	0.777–2.614	0.262	1.427	0.658–3.093	0.368
Tumour site (Rectum vs. Colon)	1.271	0.783–2.062	0.318	1.262	0.784–2.032	0.339
Tumour histology (Adenocarcinoma vs. Mucinous adenocarcinoma)	1.533	0.933–2.518	0.092	1.6	0.981–2.607	0.06
Tumour differentiation (Well/moderate vs. Poor)	1.456	1.264–1.787	0.005*	1.432	1.231–1.811	0.009*
T stage (T2/3 vs. T4)	1.54	0.92–2.58	0.105	1.68	0.95–2.78	0.219
N stage (N0 vs. N1/2)	1.149	0.728–1.897	0.472	1.234	0.843–1.942	0.573
TNM stage (III + IV vs. I+II)	2.116	1.301–3.443	0.003*	2.077	1.287–3.351	0.003*
Lymph vascular invasion (Absence vs. Presence)	1.14	0.75–1.42	0.063	1.23	0.84–1.52	0.072
Distant metastasis (Absence vs. Presence)	1.53	0.98–2.28	0.064	1.49	1.02–2.34	0.059
Expression of H19 (High vs. Low)	1.505	1.027–2.905	0.036*	1.732	1.036–2.933	0.036*
**Multivariate analysis**						
TNM stage (III + IV vs. I+II)	2.142	1.421–3.24	0.007*	2.041	1.345–3.098	0.008*
Tumour differentiation (Well/moderate vs. Poor)	1.466	1.263–1.828	0.009*	1.412	1.218–1.776	0.006*
Expression of H19 (High vs. Low)	1.521	1.303–1.896	0.018*	1.433	1.239–1.786	0.006*

### Manipulation of H19 levels in CRC cells

**Figure 3:**
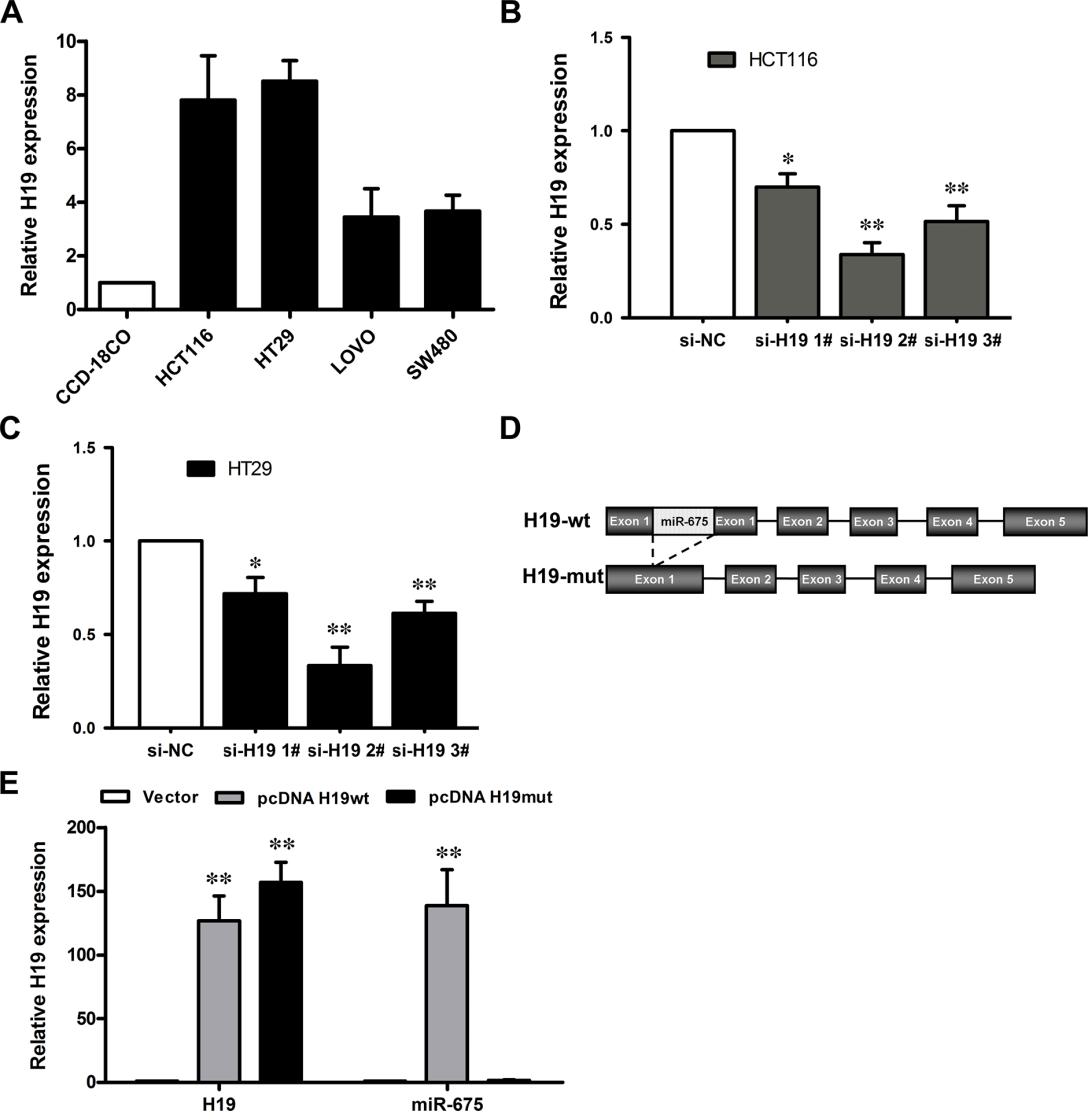
The level of H19 expression in CRC cells (**A**) Results from qRT-PCR, which demonstrate the H19 expression level of CRC cell lines (HCT116, HT29, LOVO and SW480) compared with a normal human colon epithelial cell line (CCD-18Co). (**B**, **C**) qRT-PCR analyses of the H19 expression levels following treatment in HCT116 (B) and HT29 (C) cells with si-H19 or si-NC (negative control). **P < 0.01 (**D**) Schematic diagram of H19 overexpression plasmid construction. (**E**) qRT-PCR analyses of the H19 expression level following the treatment of CCD19-Co cells with pCDNA3.1-H19 wt, pCDNA3.1-H19 mut or empty vector. The data are expressed as the mean ± SD. The results are representative of three independent experiments. *P < 0.05, **P < 0.01.

To evaluate the biological functions of H19, we next performed qRT-PCR analysis to examine the expression levels of H19 in a variety of cell lines, including HCT116, HT29, SW480, Lovo, and the normal colon epithelium cell line CCD-18Co. The results showed that H19 expression was obviously upregulated in the CRC cell lines (Figure 3A), which suggests that an increase in the expression levels could be significant in colorectal carcinogenesis.

To manipulate the H19 levels in the CRC cells, si-H19 was transfected into HCT116 and HT29 cells, and the pCDNA-H19wt and pCDNA-H19mut vectors were transfected into CCD-18Co cells. qRT-PCR analysis revealed that H19 expression was effectively knocked down in both si-H19-transfected HCT116 and HT29 cells compared with si-NC (negative control) cells (Figure 3B and 3C). H19 expression was effectively 65∼70% knocked down by si-H19-2, the most effective siRNA subsequently used in the following experiments.

Because H19 was reported to be the primary miRNA precursor of miR-675, a mutant H19 transcript vector (pCDNA-H19mut) was also developed by the deletion of the first exon and transfected into CCD-18Co cells to determine whether the function of H19 was due to miR-675 (Figure 3D). qRT-PCR analysis of H19 and miR-675 levels was performed at 48 h post-transfection and revealed that H19 expression was increased 126-fold after transfection of pCDNA-H19wt and 147-fold of pCDNA-H19mut in CCD-18Co cells, compared with control cells. Furthermore, transfection of pCDNA-H19wt could increase miR-675 expression by 138-fold, whereas the pCDNA-H19mut vector had no effect on the miR-675 expression (Figure 3E).

### Effect of H19 on CRC cell proliferation

**Figure 4:**
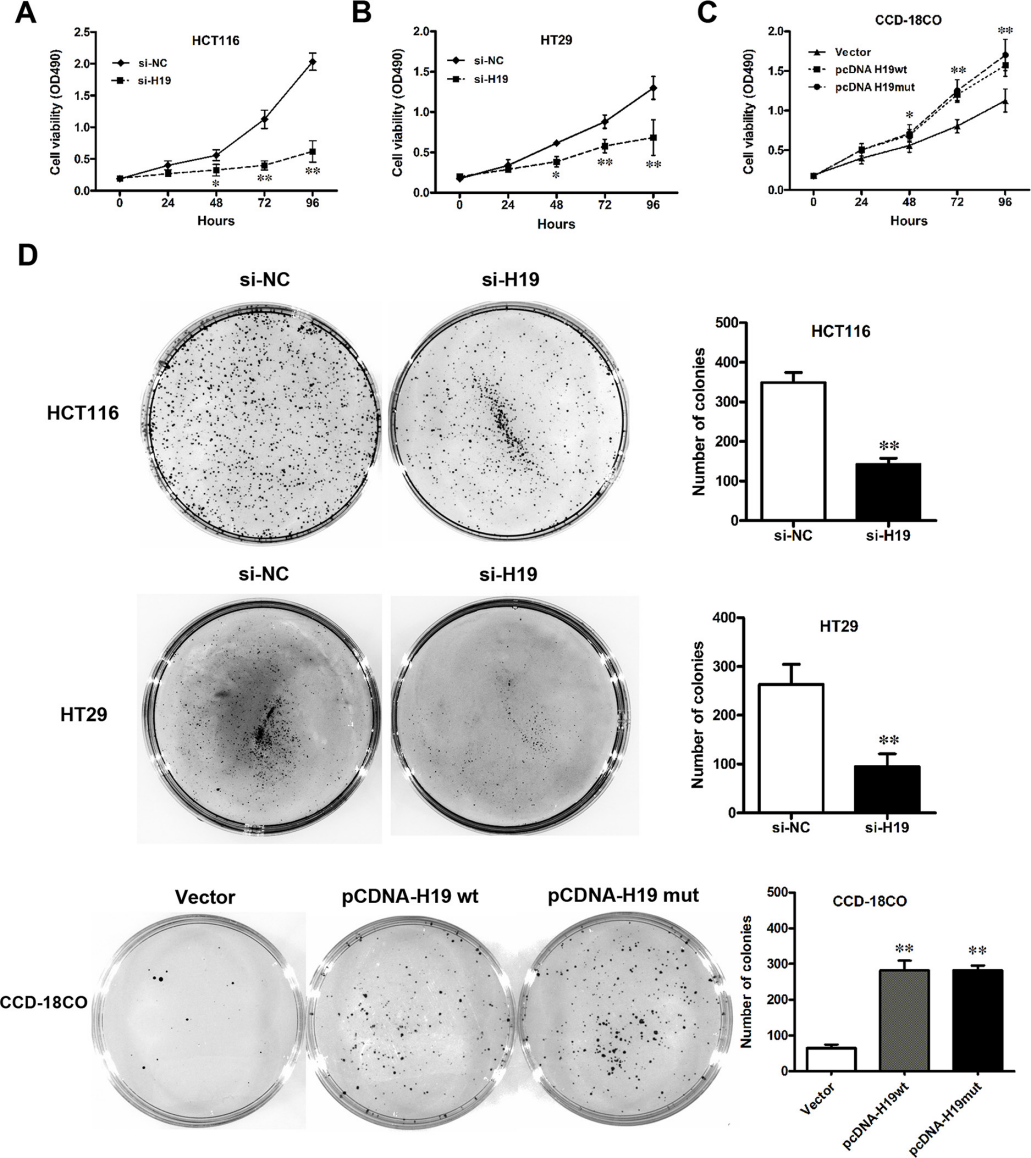
Effect of H19 on CRC cell proliferation (**A**–**C**) Cell viability of HCT116 (A) and HT29 (B) cells when H19 was downregulated; CCD-18Co (C) cells when H19 was overexpressed. (**D**) Colony formation of CRC cells with pCDNA-H19wt/mut or CCD-18Co cells with si-H19 treatment. The data are expressed as the mean ± SD. The results are representative of three independent experiments. *P < 0.05, **P < 0.01.

The significant increase in H19 expression in CRC samples prompted us to explore the possible biological significance of H19 in tumorigenesis. To assess the biological role of H19 in CRC, we investigated the effect of targeted knockdown or overexpression of H19 on cell proliferation. MTT assay and trypan blue staining revealed that the proliferation of HCT116 or HT29 cells was decreased in both si-H19-2 (Figure 4A and 4B) and si-H19-3 (Supplementary Figure 1A and 1B) transfected cells compared with the respective controls. The cell growth was significantly impaired in both pCDNA-H19wt and pCDNA-H19mut transfected CCD-18Co cells (Figure 4C). Notably, there is no obvious difference between the effects of pCDNA-H19wt and pCDNA-H19mut, which indicates that the effect of H19 on increasing CRC cell proliferation is not mediated by its encoding miR-675. Similarly, the results of colony-formation assays revealed that clonogenic survival was decreased following both si-H19-2 (Figure 4D) and si-H19-3 (Supplementary Figure 1C) transfection in HCT116 or HT29 cells, while it was enhanced in both pCDNA-H19wt- and pCDNA-H19mut-transfected CCD-18Co cells without any difference (Figure 4D). These data indicate that H19 promotes CRC cell proliferation, and this effect is not associated with its encoding miR-675.

### H19 promotes CRC cell proliferation by accelerating the cell-cycle progression

**Figure 5:**
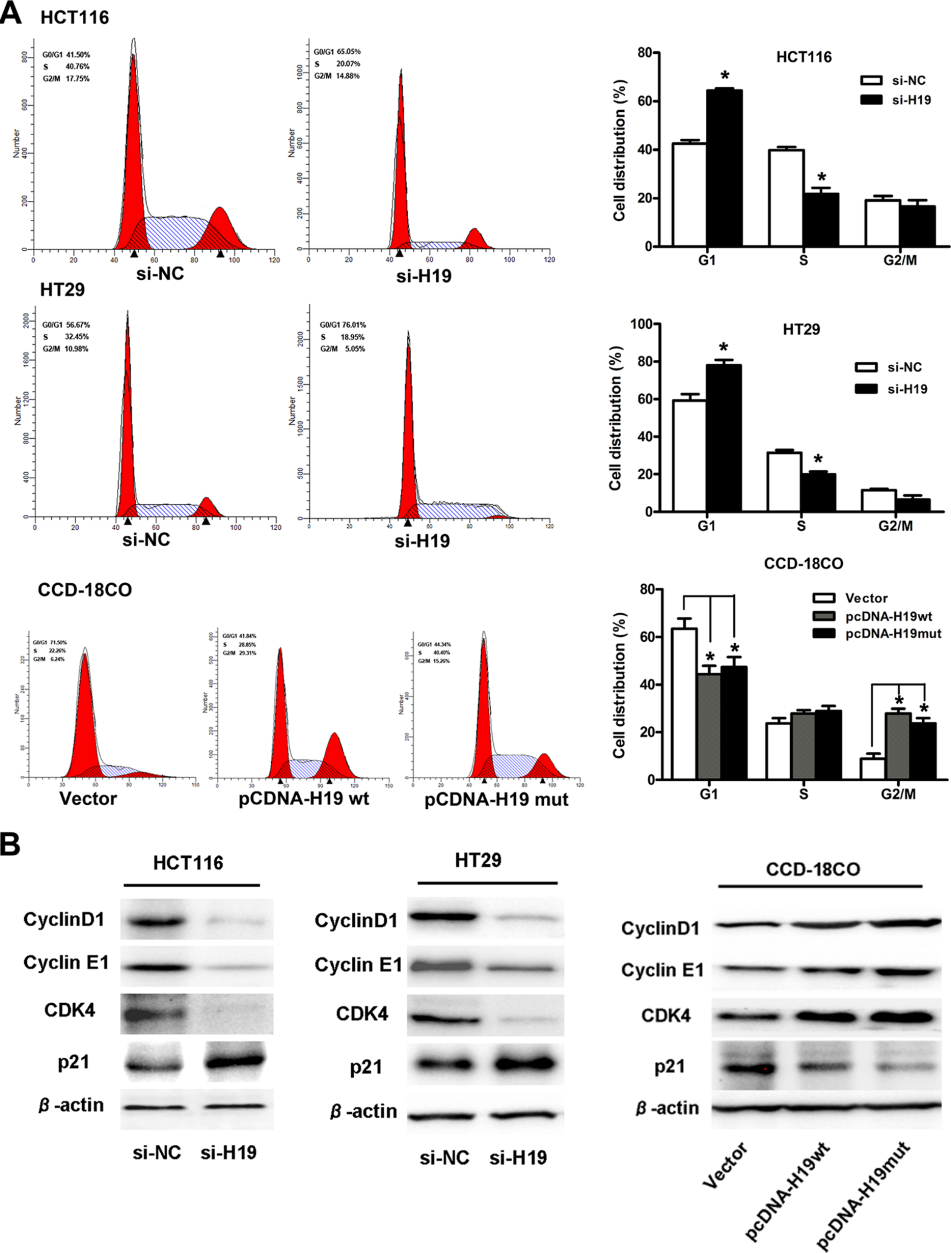
H19 promotes CRC cell proliferation by upregulating cell-cycle-regulatory genes and accelerating cell-cycle progression (**A**) Cell cycle analysis of CRC cells treated with si-H19 or CCD-19Co cells treated with pCDNA-H19wt/mut for 48 hours. The bar chart represents the percentage of cells in the G1-G0, S, or G2-M phase, as indicated. (**B**) Protein expression changes of cell-cycle-regulatory genes in CRC cells treated with si-H19 or CCD-19Co cells treated with pCDNA-H19wt/mut for 48 hours. The data are expressed as the mean ± SD. The results are representative of three independent experiments. *P < 0.05, **P < 0.01.

To further examine whether the effect of H19 on the proliferation of CRC cells occurs by altering cell-cycle progression or apoptosis, we performed flow cytometric analysis. The results revealed that the cell-cycle progression of the si-H19-transfected HCT116 or HT29 cells was significantly stalled at the G0/G1 phase compared with cells that were transfected with si-NC. In addition, the over-expression of H19 could obviously accelerate the cell-cycle progression (Figure 5A). However, H19 did not affect cell apoptosis after the transfection of either si-H19 or pCDNA-H19wt/pCDNA-H19mut (data not shown).

Next, we explored the expression changes of cell-cycle-regulatory genes after H19 treatment. The results showed that the expression of cyclin D1, cyclin E1 and CDK4 (at the protein level) was significantly decreased in CRC cells that were transfected with si-H19 compared to those transfected with si-NC. Moreover, an increased P21 protein level was also observed in cells that were transfected with si-H19 compared to those transfected with si-NC (Figure 5B). However, the mRNA expression of cyclin D1, cyclin E1, CDK4 or P21 remained unaltered in the H19-downregulated CRC cells compared with the NC controls (data not shown). At the same time, we also assayed for changes in the protein expression of cyclin D1, cyclin E1, CDK4 and P21 in pCDNA-H19wt- and pCDNA-H19mut-transfected CCD-18Co cells. As expected, when compared with vector control cells, the over-expression of both H19wt and H19mut resulted in an increase in cyclin D1, cyclin E1 and CDK4 levels and a decrease in the P21 levels (Figure 5B). These data suggest that H19 promotes CRC cell proliferation by regulating the cell-cycle-regulatory gene expression and then accelerating the cell-cycle progression.

### LncRNA-H19 combines with eukaryotic translation initiation factor 4A3 (eIF4A3)

**Figure 6:**
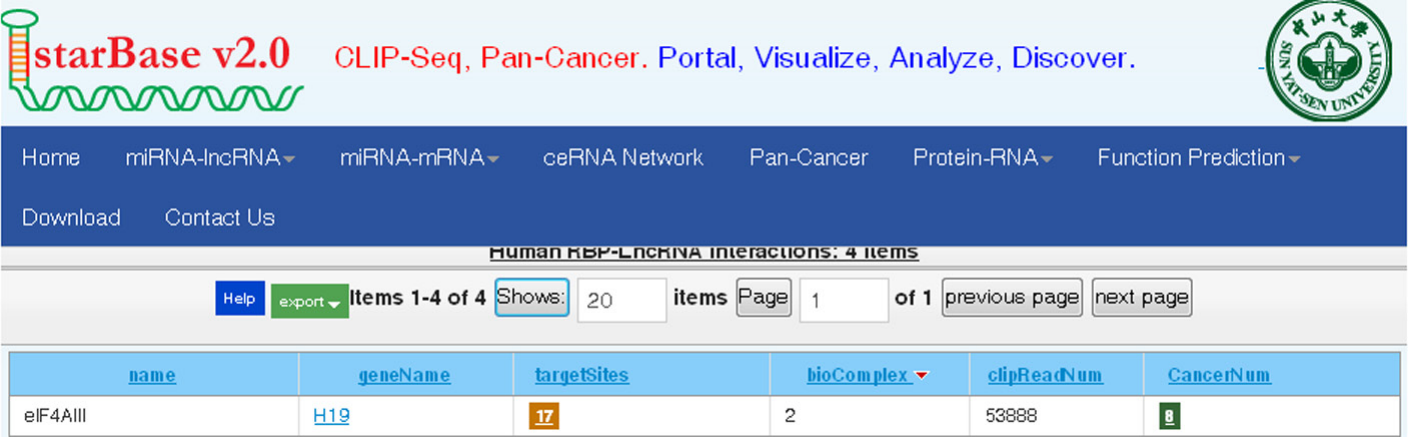
Bioinformatics prediction of H19 potential binding proteins by the starbase v2.0 database

**Figure 7:**
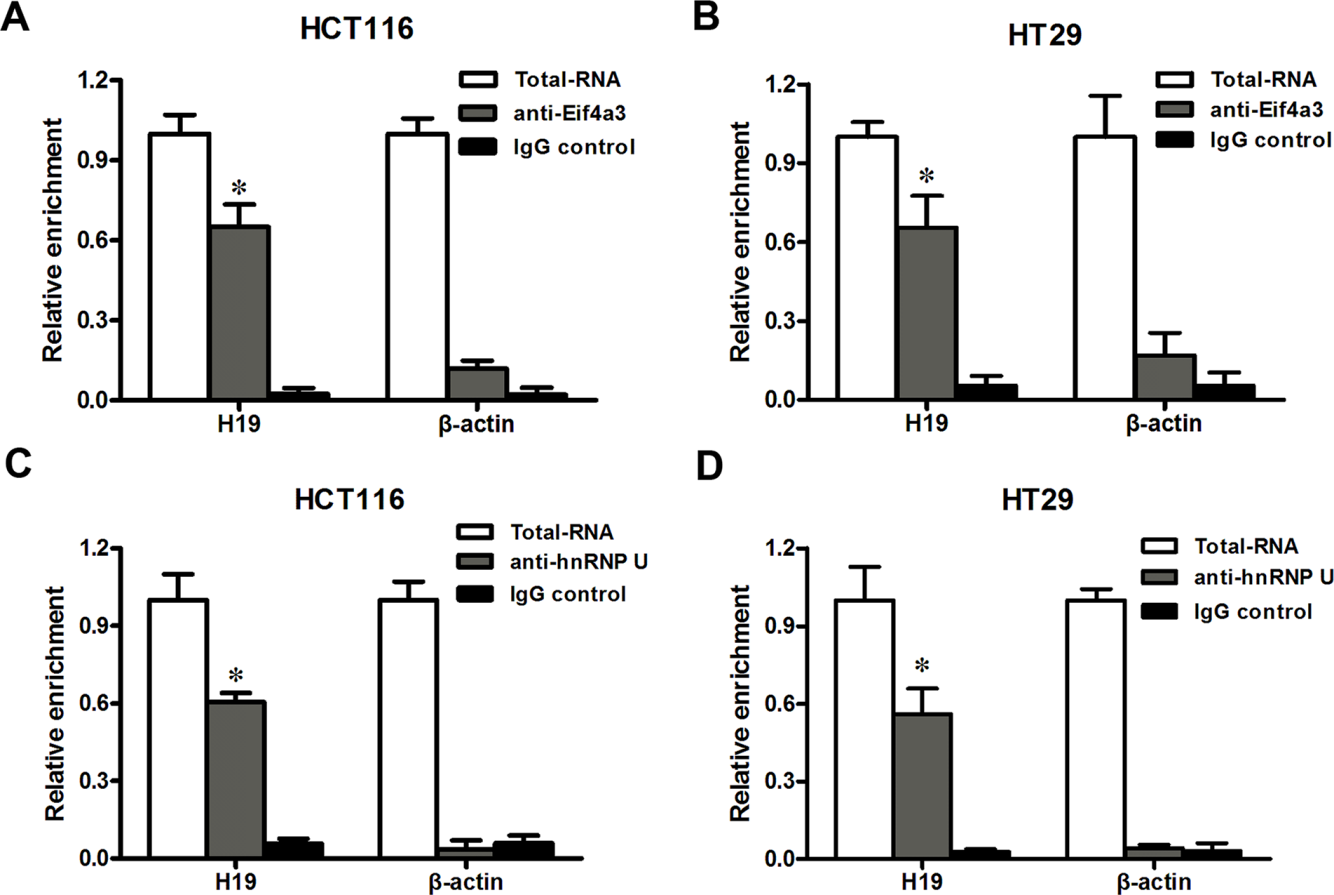
H19 could bind to eIF4A3 (**A**–**B**) RIP experiments were performed in HCT116 (A) and HT29 (B) cells, and the coprecipitated RNA was subjected to qRT-PCR for H19. The fold enrichment of H19 in eIF4A3 RIP is relative to its matching IgG control RIP. (**C**–**D**) hnRNP U was used as a positive control in HCT116 (C) and HT29 (D) cells. The data are expressed as the mean ± SD. The results are representative of three independent experiments. *P < 0.05, **P < 0.01.

In an earlier part of this study, we found that H19 promoted the growth of CRC cells and affected the expression of a group of genes that are involved in the cell cycle. This effect of H19 is difficult to explain based on the mechanisms of action that have been previously reported [14] because we did not observe participating of miR-675 in the CRC cells proliferation. Several recent studies have found that many lncRNAs participate in molecular regulation pathways through their interactions with proteins [27]. To investigate whether lncRNA-H19 functions in such a manner, we used the starBase v2.0 database to identify proteins that potentially combine with H19 (Figure 6). Of four candidate proteins predicted in the database, eIF4A3 was identified as the binding protein of H19 by RNA-binding protein immunoprecipitation (RIP) followed by qPCR in HCT116 and HT29 cells (Figure 7A and 7B). We observed H19 enrichment, but not β-actin mRNA enrichment. Moreover, hnRNP U protein, which was previously reported to bind with H19, as determined using an anti-hnRNP U antibody, was used as a positive control (Figure 7C and 7D) [28]. The above data indicate that eIF4A3 combines with H19 and could potentially participate in CRC cell proliferation.

### H19 could control cell-cycle-regulatory gene expressions by obstructing the recruitment of eIF4A3 to their mRNA

**Figure 8:**
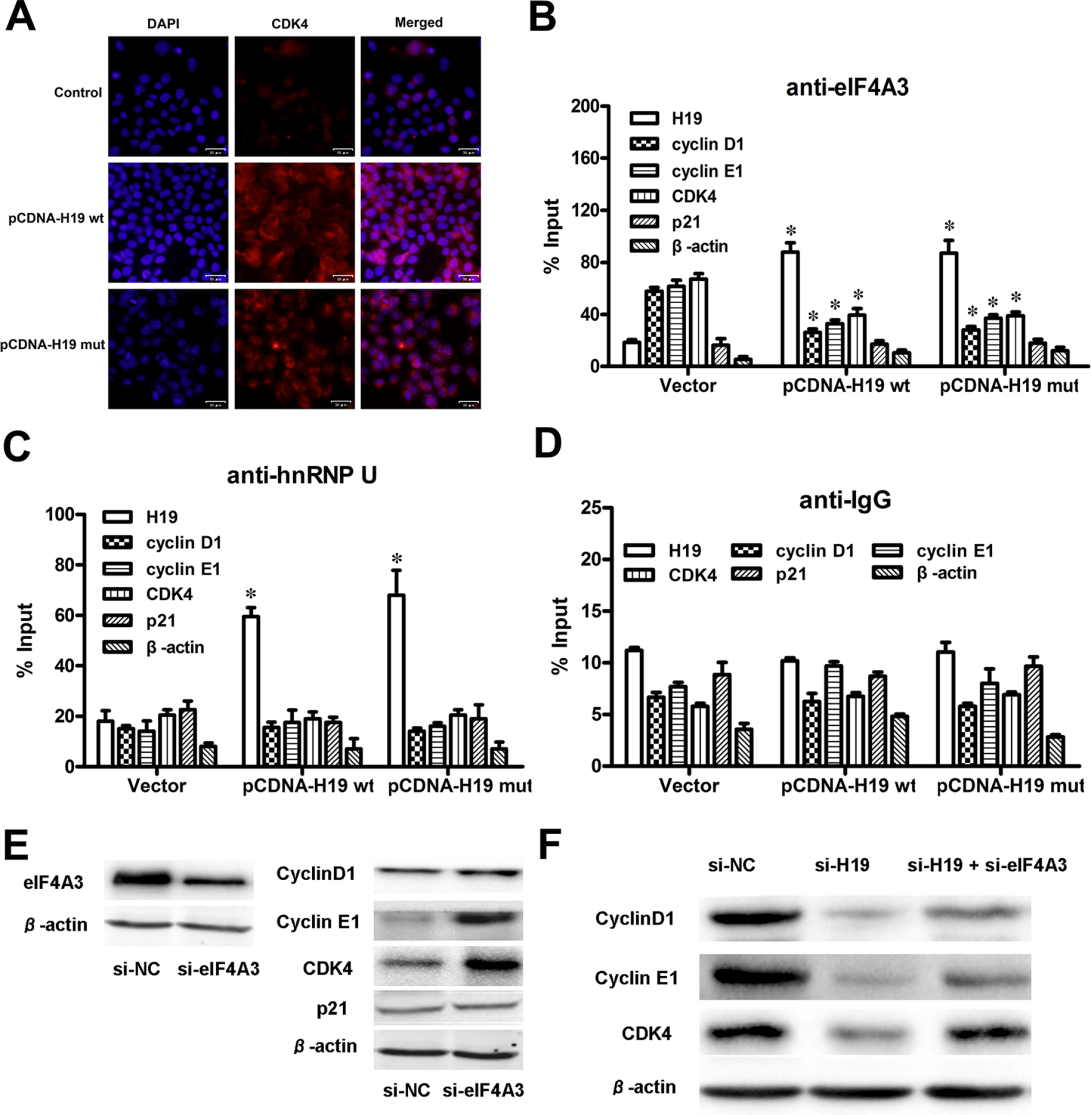
H19 could control cell-cycle-regulatory gene expressions by obstructing the recruitment of eIF4A3 to their mRNA (**A**) IF images showed CDK4 expression in HCT116 cells treated with pCDNA-H19wt/mut for 48 hours. (**B**) RIP experiments were performed using an antibody against eIF4A3 on extracts from HCT116 cells followed by H19 overexpression. The purified RNA was used for qRT-PCR analysis, and the enrichment of H19, cyclin D1, cyclin E1, CDK4, P21 and β-actin was normalized to the input. (**C**–**D**) HnRNP U (C) and IgG (D) were used as negative controls. (**E**) The protein levels of cell-cycle-regulatory genes were detected in HCT116 cells after si-eIF4A3 treatment by western blot analysis. (**F**) The comparison and quantification of cyclin D1, cyclin E1 and CDK4 proteins in HCT116 cells with or without si-eIF4A3 after si-H19 treatment. The data are expressed as the mean ± SD. The results are representative of three independent experiments. *P < 0.05, **P < 0.01.

**Figure 9:**
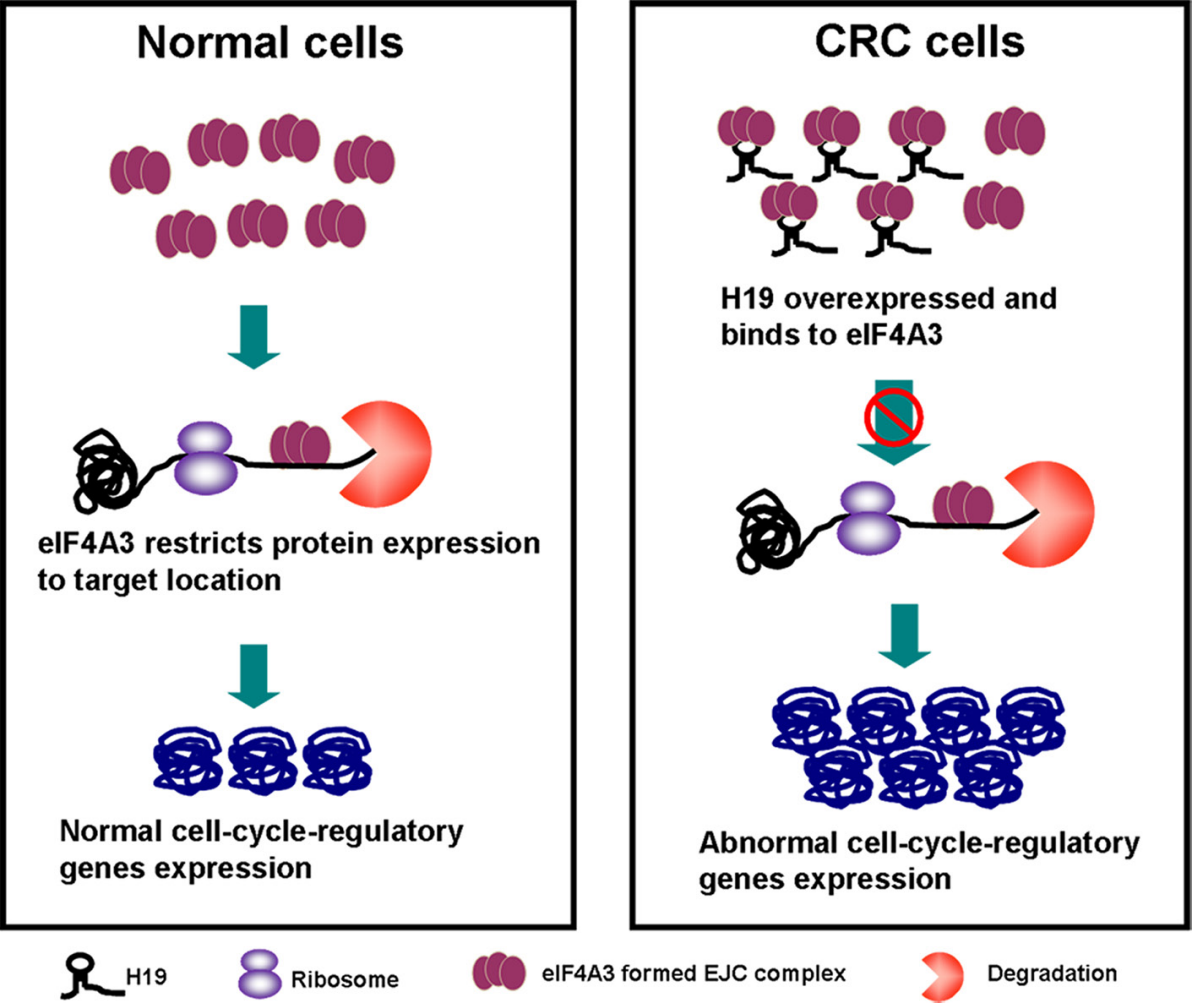
The diagram of the H19/eIF4A3 regulatory network that regulates the proliferation of CRC cells

Previously, we found that H19 upregulated the expression of cyclin D1, cyclin E1 and CDK4. The IF assay also showed that the cell count and CDK4 expression both increased in pCDNA-H19wt- and pCDNA-H19mut-treated cells (Figure 8A). We also determined that H19 potentially binded with eIF4A3. The eukaryotic translation initiation factor 4A3 (eIF4A3) is a component of the exon junction complex, which plays an important role in monitoring the mRNA quality before it progresses into a translation event. Researchers found that eIF4A3 could modulate neuronal protein expression and synaptic strength [29]. Based on these findings, we hypothesize that H19 binds to eIF4A3 and affects its abundance in the mRNA region of cyclin D1, cyclin E1 and CDK4, and then, it affects the cell-cycle-regulatory gene expressions at the translational or posttranslational levels. To identify these hypotheses, we applied the RIP assay to detect the enrichment of cyclin D1, cyclin E1 and CDK4 mRNA on eIF4A3 after the over-expression of H19. As shown in Figure 8B, cyclin D1, cyclin E1 and CDK4 mRNA was less enriched in the anti-eIF4A3 RNA immunoprecipitation fraction after the transfection of both pCDNA-H19wt and pCDNA-H19mut. Anti-hnRNP U and anti-IgG antibodies were used as negative controls (Figure 8C and 8D). These data suggest that H19 overexpression decreased the eIF4A3 abundance in the mRNA region of cyclin D1, cyclin E1 and CDK4. To further validate this mechanism, we first detect the expression of the above genes after si-eIF4A3 treatment. The results showed that the expression of cyclin D1, cyclin E1 and CDK4 was significantly increased in cells that were transfected with si-eIF4A3 compared to those transfected with si-NC (Figure 8E). Further validation of the underlying mechanisms was performed by examining the expression of these genes after transfection with si-H19 and/or si-H19 together with si-eIF4A3 or si-NC. As shown in Figure 8F, si-eIF4A3 abolished the inhibiting effect of si-H19 on the expression of cell-cycle-regulatory genes. Taken together, our data suggest that H19 obstructs the recruitment of eIF4A3 to the cell-cycle-regulatory genes mRNA, which results in the acceleration of the cell-cycle progression and CRC cell proliferation (Figure 9).

## DISCUSSION

LncRNAs are emerging from the “desert region” of the genome as a new source of biomarkers that could characterize disease recurrence and progression [30, 31]. Although most cancer-related lncRNAs have the same expression pattern and biological function, it is still important to note that some exact lncRNAs play different roles in distinct cancer types. H19 is reported to be associated with some types of carcinoma, and it is overexpressed and acts as an oncogene in breast [19, 20], bladder [23], glioma [32], and prostate [33] cancers; however, it still functions as a tumor-suppressor in hepatocellular cancer [34]. This difference could be due to different lncRNA molecular mechanisms: 1) H19 contains a microRNA (miR-675) in the first exon [35], which is responsible for its oncogenic activity by regulating the target genes of miR-675 in glioma and prostate cancer. 2) In contrast, as reported in HCC (hepatocellular cancer) [34], H19 expression was down-regulated and associated with the protein complex hnRNP U/PCAF/RNAPol II, which activates the miR-200 family by increasing the histone acetylation, thus contributing to the suppression of tumor metastasis. Because lncRNAs have been reported to be involved in all aspects of gene regulation [36], there are still significant gaps in our current understanding of lncRNA function. One exact lncRNA could have diverse biological functions in the whole genome [37]. As for H19, some of its novel functions have been uncovered recently. Report of Zeira et al. showed that H19 plays regulatory role in pluripotency and tumorigenesis of human embryonic carcinoma cells [38]. Pu and colleagues found that H19 together with another lncRNA CUDR promotes liver cancer stem cell growth through upregulating TERT and C-Myc [39]. Besides, several researches reported that H19 plays an important role in tumor metastasis by promoting epithelial to mesenchymal transition [40, 41].

In this study, we aimed to uncover the exact biological function of H19 in the tumorigenesis of CRC. We tested the expression of H19 in CRC samples and their surrounding non-tumorous tissues. We also identified the function of H19 in the CRC cells by applying gain-of-function and loss-of-function approaches. Because miR-675 is derived from the first exon of H19, a first exon deletion mutant of H19 was also used to examine whether H19 has other functions besides encoding miR-675. The results demonstrated that H19 was upregulated in CRC tissues in comparison with adjacent normal colon tissue and that H19 upregulation correlated with tumor differentiation and TNM stage. Moreover, the overall and disease-free survival time of patients with lower H19 expression levels was significantly longer than that of patients with moderate or strong H19 expression levels. Furthermore, H19 overexpression promoted the proliferation of CRC cells by accelerating cell-cycle progression, while H19 depletion inhibited cell viability and induced growth arrest. These findings suggest that H19 promotes CRC progression.

The importance of lncRNAs in human diseases could be associated with their ability to impact cellular function through various mechanisms [8, 42]. Previously, the oncogenic role of H19 in CRC was reported to be associated with its function as the precursor of miR-675 [14]. However, in the present study, our data show that H19mut promotes cell proliferation and upregulates cell-cycle-regulatory genes and there is no difference between the effects of H19wt, which indicates that H19 has other functions in addition to encoding miR-675. The pathological role of H19 in CRC is difficult to explain by the H19/miR-675 mechanism, which has been reported in prostate and glioma carcinoma cells. Recently, Li and colleagues uncovered some functional relationship between H19 and miR-675 [43]. Their work showed that miR-675 enhanced H19 transcription and accelerated hepatocarcinogenesis through H19-target genes, which stimulated us to reveal the biological mechanism of H19 in CRC carcinogenesis. Several recent studies have found that many lncRNAs participate in molecular regulation pathways through their interactions with proteins [27]. We performed bioinformatics prediction and RIP analysis to identify and validate proteins that are potentially combined with H19. As a result, eIF4A3 was identified as the binding protein of H19.

EIF4A3 and three other proteins constitute the core of a larger assemblage known as the exon junction complex (EJC) [44–46]. Within this core, the DEAD-box protein eIF4A3 collaborates with its binding partner MLN51 to serve as the main RNA binding constituent [47]. EIF4A3-formed EJC can trigger nonsense-mediated mRNA decay [48] and modulate protein expression at the translational and post-translational levels. Giorgi and colleagues [29] showed that eIF4A3 knockdown markedly increased both the synaptic strength and GLUR1 AMPA receptor abundance at synapses. Based on these results, we hypothesize that H19 binds to eIF4A3 and affects eIF4A3 abundance in the mRNA region of cyclin D1, cyclin E1 and CDK4, and then, it affects the cell-cycle-regulatory gene expressions at the translational or post-translational level. RIP assay followed by H19 overexpression was used to validate our hypothesis, and the results showed that cyclin D1, cyclin E1 and CDK4 mRNA were less enriched in the anti-eIF4A3 RNA immunoprecipitation fraction after the transfection of both pCDNA-H19wt and pCDNA-H19mut. However, we did not detect any changes in the mRNA region of P21, which could be because different mRNA regions of the eIF4A3-formed EJCs that were located could have distinct consequences [47]. Further experiments are needed to elucidate the exact mechanism. In the present study, we also enforced cyclin D1, cyclin E1 and CDK4 expression by using only si-eIF4A3 or bidirectional si-H19 and si-eIF4A3 together and found that an inhibition effect of si-H19 on cyclin D1, cyclin E1 and CDK4 expression was deprived by the induction of si-eIF4A3 (Figure 9).

In conclusion, our present work uncovers a novel H19-mediated mechanism of cell cycle regulation in CRC cells. Although further studies are required to clarify H19/eIF4A3 regulation of P21 expression, the H19/eIF4A3/cyclin D1/E1/CDK4 pathway was still shown to be involved in the development of CRC, and targeting this pathway might have therapeutic potential for CRC. Other lncRNAs that are differentially expressed in CRC and adult colons should also be explored to determine whether they play significant roles in CRC tumorigenesis.

## MATERIALS AND METHODS

### Tissue samples and clinical data collection

A total of 83 patients analyzed in this study underwent resection of the colorectal cancer at the Second Affiliated Hospital of Harbin Medical University. The study was approved by the Research Ethics Committee of Harbin Medical University (Harbin, Heilongjiang, PR China), and written informed consent was obtained from all of the patients. The clinicopathological characteristics of the CRC patients are summarized in Table 1. Follow-up studies included physical examination, laboratory analysis, and computed tomography if necessary. The overall survival (OS) was defined as the interval between the dates of surgery and death. Disease-free survival (DFS) was defined as the interval between the dates of surgery and recurrence; if recurrence was not diagnosed, then the patients were censored on the date of death or the last follow-up.

### Cell culture

Four CRC cell lines (HCT116, HT29, Lovo, SW480) and a normal colon epithelium cell line (CCD-18Co) were purchased from the the American Type Culture Collection (ATCC, Manassas, VA, USA). The cells were cultured in RPMI 1640 or DMEM (GIBCO-BRL) medium supplemented with 10% fetal bovine serum (10% FBS), 100 U/ml penicillin, and 100 mg/ml streptomycin in humidified air at 37°C with 5% CO2.

### RNA extraction and qRT-PCR analyses

The total RNA was extracted from the tissues or cultured cells using TRIzol reagent (Invitrogen, Carlsbad, CA). Reverse transcribed complementary DNA was synthesized with random primers or microRNAs specific stem-loop primers. Subsequently, the cDNA was subjected to real-time PCR on a 7500 real-time PCR system (AB Applied Biosystems, Mannheim, Germany). β-actin and U6 were used as internal controls. The PCR primers were as follows:

H19: F: 5′-CTTCTGGGCTCAAGTGATCCT-3′, R: 5′-TTGTGCCATGAGACTCCATCAG-3′; β-actin: F: 5′-GTCAACGGATTTGGTCTGTATT-3′, R: 5′-AGTCTT CTGGGTGGCAGTGAT-3′; Cyclin D1: F: 5′-GCTGCGAA GTGGAAACCATC-3′, R: 5′-CCTCCTTCTGCACACA TTTGAA-3′; Cyclin E1: F: 5′-GCCGCAGTATCCCCAG CAAA-3′, R: 5′-TCGCACCACTGATACCCTGA-3′; CD K4: F: 5′-CTCTCTAGCTTGCGGGCCTGTA-3′, R: 5′-CA AGGGAGACCCTCACGCCGA-3′; P21: F: 5′-CAGAGG AGGCGCCATGT-3′, R: 5′-GGAAGGTAGAGCTTGGG CAG-3′; miR-675: F: 5′-TGGTGCGGAGAGGGC-3′, R: 5′-GAACATGTCTGCGTATCTC-3′; U6: F: 5′-GCTTCG GCAGCACATATACT-3′ and R: 5′-GGAACGCTTCAC GAATTTGC-3′.

### Ectopic expression and knockdown of H19/H19mut

Based on the expression of H19 in the CRC cell lines, we selected CCD-18Co cells for the enhanced expression study and HCT116 and HT29 cells for the knock-down study. For ectopic expression, the full-length H19 cDNA or H19 cDNA with a deletion mutation of its first exon were subcloned into the pCDNA3.1 vector (Gene Pharma Company, Shanghai, China) and transfected into CCD-18Co cells. For the knock-down of H19 or eIF4A3 expression, the nucleotide sequences of siRNA (H19-1-CCAACAUCAAAGACACCAU, H19-2-GCAGGACAUGACAUGGUCC, H19-3-UAAG UCAUUUGCACUGGUU, eIF4A3-AGACAUGACUAA AGUGGAA) were chemically synthesized and transfected into HCT116/HT29 cells. Briefly, a total of 1.2 × 10^5^ cells were plated in 6-cm culture dishes overnight and then transfected with the vectors or siRNAs described above using Lipofectamine 2000 (Invitrogen) for 48 h. The negative control (Gene Pharma Company, Shanghai, China) was transfected in parallel. The cells were then subjected to RNA/protein extraction and further functional assays.

### Cell proliferation assays

Non-transfected or transfected cells were re-seeded into 96-well plates (5 × 10^3^ cells/well). Cell viability was tested using an MTT kit (Sigma Chemicals, St Louis, MO, USA) according to the manufacturer's instructions. The absorbance of each well at the wavelength of 492 nm was read on a spectrophotometer. At least three independent experiments were performed in quadruplicate. For a colony formation assay, approximately 500–800 transfected cells were placed in each well of the 6-well plates and maintained in proper media that contained 10% FBS for 7–10 weeks, during which the medium was replaced every 2 days. Colonies were stained with 0.1% crystal violet in 20% methanol for 15 min. The samples were photographed and the numbers of visible colonies were counted.

### Flow cytometric analysis

The cells were harvested, fixed in 80% ethanol and stored at 4°C. On the day of analysis, the cells were washed and centrifuged using cold PBS, and they were suspended in 10 ml of PBS and 1ml of RnaseA solution (250 mg/ml) followed by incubation for 30 minutes at 37°C. Then, 100 μl of propidium iodide stain (100 mg/ml) was added to each tube, which was then incubated at 4°C for 30 minutes prior to analysis. Flow cytometric analysis was conducted using BD LSRFortessa (BD Biosciences). The percentages of the cells in different phases of the cell cycle were analyzed by using FlowJo software.

### Western blot assay and antibodies

Cellular protein extracts were separated in a 12 or 8% SDS-polyacrylamide gel and electrophoretically transferred onto a PDVF membrane (Millipore, Bedford, MA, USA). Membranes were blocked overnight with 5% non-fat dried milk and incubated with antibodies to cyclin D1/cyclin E1/CDK4/P21 (Cell Signaling Technology, Danvers, MA, USA, 1:1000), eIF4A3/hnRNP U (Abcam Biotechnology, USA, 1:1000) or Beta-actin (Santa Cruz Biotechnology, Santa Cruz, CA, USA, 1:1000) overnight at 4°C. After washing with PBST, the membranes were incubated with horseradish peroxidase-linked secondary antibody. The proteins were visualized using ECL chemiluminescence and exposed to X-ray film. Bands were quantified with Image J (National Institutes of Health, Bethesda, MD, USA).

### Immunofluorescence (IF) analysis

The cells were rinsed with PBS and fixed with 4% paraformaldehyde for 30 minutes at room temperature, followed by permeabilization with 0.1% sodium citrate plus 0.1% Triton X-100. The cells were subjected to immunofluorescent staining with CDK4 antibody (1:200) for 16 hours at 4°C. The cells were then washed with cold PBS three times for five minutes each and incubated with fluorescence-labeled secondary antibody (1:400, #ZF0511, ZSGB-BIO) for 30 minutes. The cells were visualized using an inverted fluorescence microscope (FSX100, Olympus).

### RNA immunoprecipitation (RIP)

RNA immunoprecipitation (RIP) experiments were performed using a Magna RIP™ RNA-Binding Protein Immunoprecipitation Kit (Millipore, USA) according to the manufacturer's instructions. The antibodies used for RIP assays of eIF4A3 and hnRNP U were obtained from Abcam Company.

### Statistical analysis

Data were presented as mean ± standard deviation (s.d.) from at least three separate experiments. The Student's *t*-test, χ^2^ test or Fisher's exact test was used for comparisons between groups. The DFS and OS rates were calculated using the Kaplan-Meier method, with the log-rank test applied for comparison. Survival data were evaluated using the univariate and multivariate Cox proportional hazards model. Variables with a value of *p* < 0.05 in univariate analysis were used in subsequent multivariate analysis on the basis of Cox regression analyses. Two-sided *p*-values were calculated, and a probability level of 0.05 was chosen for statistical significance.

## SUPPLEMENTARY FIGURE


