# Supplementary material for: Long noncoding RNA H19 indicates a poor prognosis of colorectal cancer and promotes tumor growth by recruiting and binding to eIF4A3
Source: Oncotarget. 2016 Mar 14;7(16):22159–73. doi: 10.18632/oncotarget.8063 (PMC5008352; doi:10.18632/oncotarget.8063)
Supplement: Supplementary file 1 [file oncotarget-07-22159-s001.pdf]

Long noncoding RNA H19 indicates a poor prognosis of colorectal cancer and promotes tumor growth by recruiting and binding to eIF4A3

Supplementary Materials

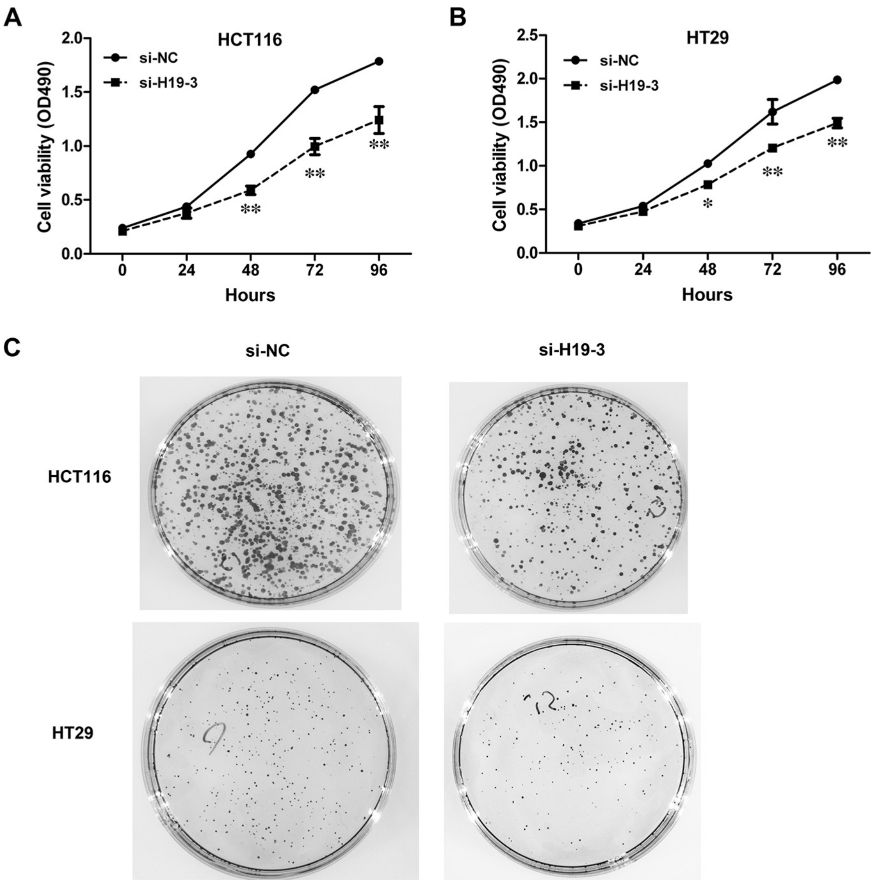

Supplementary Figure S1: The si-H19-3 inhibited CRC cell proliferation effectively. Cell viability of HCT116 (**A**) and HT29 (**B**) cells with si-H19-3 treatment. (**C**) Colony formation of HCT116 and HT29 cells with si-H19-3 treatment. The data are expressed as the mean ± SD. The results are representative of three independent experiments. $^*P < 0.05$, $^{**}P < 0.01$.
